# Serial Plasma Comprehensive Genomic Profiling Captures Therapy Resistance and Guides Management of Non–Small Cell Lung Cancer

**DOI:** 10.1158/2767-9764.CRC-25-0561

**Published:** 2026-05-21

**Authors:** Michael Conroy, Jaime Wehr, Vivian V. Altiery De Jesus, Archana Balan, William Bowers, Jennifer W. Li, Susan C. Scott, Benjamin Levy, Kristen A. Marrone, Vincent K. Lam, Josephine Feliciano, Christine L. Hann, Aliyah Pabani, Jarushka Naidoo, Julie R. Brahmer, Patrick M. Forde, Valsamo Anagnostou, Joseph C. Murray

**Affiliations:** 1 Sidney Kimmel Comprehensive Cancer Center, Johns Hopkins University School of Medicine, Baltimore, Maryland.; 2 The Zanvyl Krieger School of Arts and Sciences, Johns Hopkins University, Baltimore, Maryland.; 3 Beaumont RCSI Cancer Centre, Dublin, Ireland.; 4 The Lung Cancer Precision Medicine Center of Excellence, Johns Hopkins University School of Medicine, Baltimore, Maryland.

## Abstract

Plasma comprehensive genomic profiling (pCGP) is implemented in the clinical care of non–small cell lung cancer (NSCLC). Although less well documented, serial pCGP may also guide the management of oncogene-driven NSCLC after first progression. In this study, we assessed the clinical value of serial pCGP, focusing on *EGFR*-mutant NSCLC. We conducted a retrospective study of 718 patients with NSCLC who underwent pCGP between 2015 and 2022. Clinical genomic data were programmatically extracted from the data workflows of the Johns Hopkins Lung Cancer Precision Medicine Center of Excellence. After variant annotation and actionability characterization, we examined the prevalence and evolving comutation patterns across serial pCGP, focusing on genomic mechanisms of tyrosine kinase inhibitor (TKI)–acquired resistance in *EGFR*-mutant NSCLC. A total of 718 patients had 818 instances of pCGP, with 79 patients having longitudinal pCGP (range, 2–5). pCGP uniquely informed management in 13% of patients (*n* = 92), both at initial diagnosis and on serial genotyping. This occurred predominantly through the identification of actionable mutations when tissue testing was unavailable. Among 214 patients with *EGFR*-mutant NSCLC, pCGP identified PI3K pathway alterations in 11% after first-line therapy. *BRAF* V600E (3%) and *MET* exon 14 skipping mutations (3%) emerged after third-generation TKI therapy. After TKI progression, 31 patients (22%) with *EGFR*-mutant disease had actionable pCGP findings, of whom 18 (58%) were matched to targeted therapy. Serial pCGP can inform treatment decisions in patients with NSCLC. In those with *EGFR*-mutant disease, pCGP at progression identifies actionable drivers of therapy resistance, enabling therapeutic intervention.

**Significance:**

Our study provides critical insights into the routine implementation of serial pCGP within a thoracic oncology program, supported by a precision oncology informatics framework, in a tertiary healthcare institution. We show that pCGP enables genotyping when tissue testing is not feasible and identifies actionable mutations at resistance. The clinical implementation of pCGP can drive improved clinical outcomes by matching patients with effective interventions in a timely and minimally invasive manner.

## Introduction

Non–small cell lung cancer (NSCLC) is increasingly understood as a heterogeneous disease, in part driven by targetable genomic alterations and requiring a tailored strategy for each patient (1). To this end, comprehensive genomic profiling of NSCLC has revealed oncogene dependencies and therapeutic vulnerabilities that have been translated into treatment strategies with a clear survival benefit (2, 3). In tandem, continued advances in plasma comprehensive genomic profiling (pCGP) of circulating tumor DNA (ctDNA) support the value of ctDNA genotyping in cancer diagnosis and molecular subtyping (4–6). There is also growing evidence of its potential benefit in monitoring treatment response and the detection of minimal residual disease (7–14). pCGP can increase rates of guideline-concordant targeted therapy and reduce the time to initiation of treatment (15–19). This has translated into significantly better clinical outcomes, with improved overall survival (OS) among patients receiving therapy matched to ctDNA genomic alterations (20). In addition to genotyping, the kinetics of ctDNA may be predictive of survival in patients with *EGFR*-mutated (*EGFR*m) NSCLC on EGFR tyrosine kinase inhibitors (TKI; refs. 21, 22). Mechanisms of acquired resistance to targeted therapies may also be captured by pCGP, particularly in the context of *EGFR*m NSCLC (23–25).

However, the value of pCGP remains incompletely documented, with uncertainties about the clinical impact of serial use and its role in guiding management at the time of progression. In addition, the evolution and diversity of TKI resistance mechanisms as patients progress through different lines of TKI therapy are less well studied in the real-world setting. By leveraging the infrastructure and data workflows of the Johns Hopkins Lung Cancer Precision Medicine Center of Excellence (JH Lung Cancer PMCOE), we studied the prevalence and evolving comutation patterns of actionable alterations in serial pCGP to assess the clinical utility of pCGP compared with tissue next-generation sequencing (NGS). We specifically focused on *EGFR*m NSCLC and genomic mechanisms of TKI-acquired resistance to understand the impact of pCGP on the management of NSCLC.

## Materials and Methods

### Overall study design

This was a single-center retrospective study of patients with NSCLC who had undergone at least one pCGP at our institution, regardless of stage, treatment modality, or driver oncogene status, between 2015 and 2022. The cohort of patients, associated pCGP findings, demographic, clinicopathologic, and treatment data were programmatically retrieved from the JH Lung Cancer PMCOE database. Electronic medical records were manually reviewed to augment the curation of detailed clinicopathologic features and to determine clinical response and outcomes. The data cutoff for this study was October 31, 2022.

### JH Lung Cancer PMCOE

The JH Lung Cancer PMCOE uses the Precision Medicine Analytics Platform, an integrated data lake and analytic platform built on Microsoft Azure and Databricks. Clinical genomic sequencing data—including pCGP and tissue NGS—are integrated with demographic, clinicopathologic (e.g., diagnosis codes and pathologic reports), treatment, response (e.g., imaging reports), and outcomes (death data) from the electronic health record into the data lake. These data are transformed and projected into a secure analytic environment as a structured query language (SQL)–compatible database, with supporting data processing and statistical software. The JH Lung Cancer PMCOE operates under Institutional Review Board approval (IRB00303251).

### NGS

The pCGP platforms used were Guardant360 (*n* = 572 samples) and Guardant360 CDx (*n* = 246 samples). Blood samples were collected and analyzed by a Clinical Laboratory Improvement Amendments (CLIA)–approved assay as part of standard-of-care ctDNA genotyping. Guardant360 is a hybrid-capture NGS assay for detecting single-nucleotide variants (SNV) and insertion/deletion (indel) variants in 74 genes, amplifications in 18 genes, fusions in 6 genes, and microsatellite instability. Guardant360 CDx is a hybrid-capture NGS assay that detects SNVs and indels in 55 genes, copy-number amplifications in 2 genes, and fusions in 4 genes (Guardant Health Inc.). The limit of detection for SNVs in Guardant360 is 0.25% variant allele frequency with an input cfDNA of 10 ng. The limit of detection for SNVs in Guardant360 CDx is 1.8% with an input cfDNA of 5 ng and 0.2% with an input cfDNA of 30 ng (26). cfDNA tumor fraction estimates were not computed by the liquid biopsy assays used in this study. Tissue NGS data were extracted from available testing performed in the context of routine clinical care. These included a variety of institutional and commercially available CLIA-tested fixed-gene panel hybrid-capture NGS assays. For the concordance analyses between concurrent tissue biopsy and pCGP at the time of disease progression, we included all patients with pCGP within 8 weeks of tissue biopsy.

### Mutation characterization

Variants were tiered using the Association of Molecular Pathology, American Society of Clinical Oncology, and College of American Pathologists joint guidelines (27). Each tier 1 variant, defined as an FDA-recognized predictor of response to approved NSCLC drugs, was classified as actionable. Mutation oncogenicity characterization was extracted from OncoKB (28, 29). For a subset of emergent *EGFR* mutations, oncogenicity was derived from an ensemble approach utilizing the meta-annotator OpenCravat (30) to parse resources, including knowledge bases (e.g., ClinVar), variant registries (e.g., gnomAD), and computational annotators (e.g., FATHMM-XF, CHASMplus, REVEL). Disruptive mutations in *TP53* were defined as follows: (i) all sequence alterations that introduced a stop codon or (ii) any sequence alteration that occurred within the L2 or L3 binding domains (codons 163–195 or 236–251) and resulted in amino acid substitution with differential polarity/charge (31).

### Statistical analysis

Categorical data were summarized as frequencies with percentages and continuous data as medians and ranges. Survival was calculated using the Kaplan–Meier method, and *P* values less than a prespecified alpha of 0.05 were considered significant. Statistical analysis was completed using SPSS version 29 (IBM) and R version 4.1.0 (R Foundation for Statistical Computing). Graphs were created with R 4.1.0 using R packages ggplot, webr, and ggsankey.

## Results

### Description of the JH Lung Cancer PMCOE study cohort

We evaluated 818 instances of pCGP for 718 patients with NSCLC; demographic characteristics are summarized in Table 1. Clinical and genomic data were programmatically extracted within the data framework and workflows of the JH Lung Cancer PMCOE (“Materials and Methods”). pCGP was performed using the Guardant360 (*n* = 572, 70%) and Guardant360 CDx assays (*n* = 246, 30%; “Materials and Methods”). Seventy-nine patients (11%) had more than one instance of pCGP (range, 2–5 pCGP assays). When each patient’s first genotyping was considered, most were in the context of the initial diagnosis of NSCLC (*n* = 427, 59%), whereas 28% (*n* = 199) were at metastatic progression and 8% (*n* = 59) were at first recurrence after resection. In 5% of cases (*n* = 33), liquid biopsy was obtained due to equivocal progression, which was subsequently found to be stable disease, clinical deterioration without objective progression, or repeat workup during second opinions (collectively marked as “other” and explicitly described in Supplementary Table S1).

**Table 1.** Characteristics of study population.

Characteristic	Overall population (*N* = 718)	*EGFR*-driven subset^a^ (*N* = 214)
Median age (range), year	67 (24–95)	65 (30–95)
Female, *n* (%)	421 (59)	144 (67)
Race, *n* (%)	​	​
White	451 (63)	120 (56)
Black	139 (19)	24 (11)
Asian	77 (11)	47 (22)
Other	51 (7)	23 (11)
Smoking history, *n* (%)	​	​
Current	47 (6)	4 (2)
Ex	357 (50)	60 (28)
Never	314 (44)	150 (70)
Pathology, *n* (%)	​	​
Adenocarcinoma	674 (94)	211 (99)
Squamous cell	25 (3)	3 (1)
Other	19 (3)	0 (0)
Stage at diagnosis, *n* (%)	​	​
I	3 (1)	0 (0)
II	7 (1)	2 (1)
III	42 (6)	5 (2)
IV	663 (92)	207 (97)
Context of first plasma genotyping, *n* (%)	​	​
First diagnosis	427 (59)	94 (44)
Recurrence	59 (8)	7 (3)
Progression	199 (28)	106 (50)
Other^b^	33 (5)	7 (3)
Instances of plasma genotyping, *n* (%)	​	​
1	639 (89)	162 (76)
2	62 (9)	40 (19)
≥3	17 (2)	12 (5)
Previous treatments, *n* (%)	​	​
RT	228 (32)	78 (36)
Platinum chemotherapy	186 (26)	49 (23)
Immunotherapy	95 (13)	13 (6)
Targeted therapy	156 (22)	115 (54)
First-line targeted therapy, *n* (%)^c^	​	​
Osimertinib	49 (31)	48 (42)
Erlotinib	49 (31)	47 (41)
Afatinib	19 (12)	18 (16)
Crizotinib	14 (9)	NA
Alectinib	11 (7)	NA
Gefitinib	2 (1)	2 (2)
Other	12 (8)	NA

Demographic, pathologic, and treatment characteristics of the overall study population and of the *EGFR*-driven subset. Two patients who received erlotinib and one patient who received erlotinib did not harbor documented *EGFR* activating mutations and were as such excluded from the *EGFR*-driven subset. Other therapies included poziotinib, larotrectinib, amivantamab, and capmatinib.

Abbreviations: NA, not applicable; RT, radiotherapy.

a The *EGFR*-driven subset does not include *EGFR* exon 20 mutations.

b Details of other contexts for pCGP are listed in Supplementary Table S1.

c The denominators are *n* = 156 patients in the overall population that received any line of targeted therapy and *n* = 115 in the *EGFR*-driven subset that received any line of EGFR-targeted therapy.

### Landscape of alterations detected in pCGP

When analysis was limited to each patient’s first instance of pCGP, a total of 2,612 alterations were identified (Supplementary Fig. S1; Supplementary Table S2). Based on FDA approvals at the time of data analysis (May 2024), 269 of 718 patients (37%) had an alteration associated with an FDA-approved targeted therapy on the first instance of pCGP (*EGFR* exon 19 deletion, *EGFR* L858R mutation, *ALK* rearrangement, *KRAS* G12C mutation, *MET* exon 14 skipping mutation, *BRAF* V600E mutation, *ERBB2* exon 20 insertion, *ROS1* rearrangement, *RET* rearrangement, *NTRK* rearrangement; Supplementary Fig. S2). There were 102 instances of pCGP with no variants identified (12% of all tests performed).

To account for the impact of prior therapies, we analyzed liquid biopsies at the time of first cancer diagnosis (*n* = 427). Among these 427 patients tested, 53 (12%) had no variants identified. Approximately a third of this subset of patients, 153 of the 427 patients (36%), had an alteration identified on pCGP associated with an FDA-approved targeted therapy at the time of review. When amplifications in *MET* and *ERBB2* were included, the number of patients increased to 161 (38%). Alterations frequently occurred in *EGFR* (17% of 427 patients) and *KRAS* (G12C; 7%; Supplementary Fig. S3). Seventeen cases harbored oncogenic *RB1* alterations (*RB1* S842fs, K279*, and L512fs), and in three cases, these alterations were contemporaneous with or preceded biopsy confirmation of small cell transformation. When analysis was limited to alterations for which a genotype-matched therapy was FDA-approved in the first-line setting, 113 patients had such an alteration at first diagnosis (26%). Of these 113 patients, 96 (85%) commenced a genotype-matched targeted therapy within 30 days of pCGP results (Supplementary Table S3). Of the 93 patients with canonical activating alterations in *EGFR* or *ALK* on pCGP at first diagnosis, 86 (93%) received a matched, FDA-approved targeted therapy within 30 days of the pCGP assay. These findings reaffirm that pCGP has a high diagnostic yield for therapeutic biomarkers at the time of first diagnosis, even if they are not immediately actionable in the first-line setting.

### Impact of pCGP on clinical management

**Figure 1.**
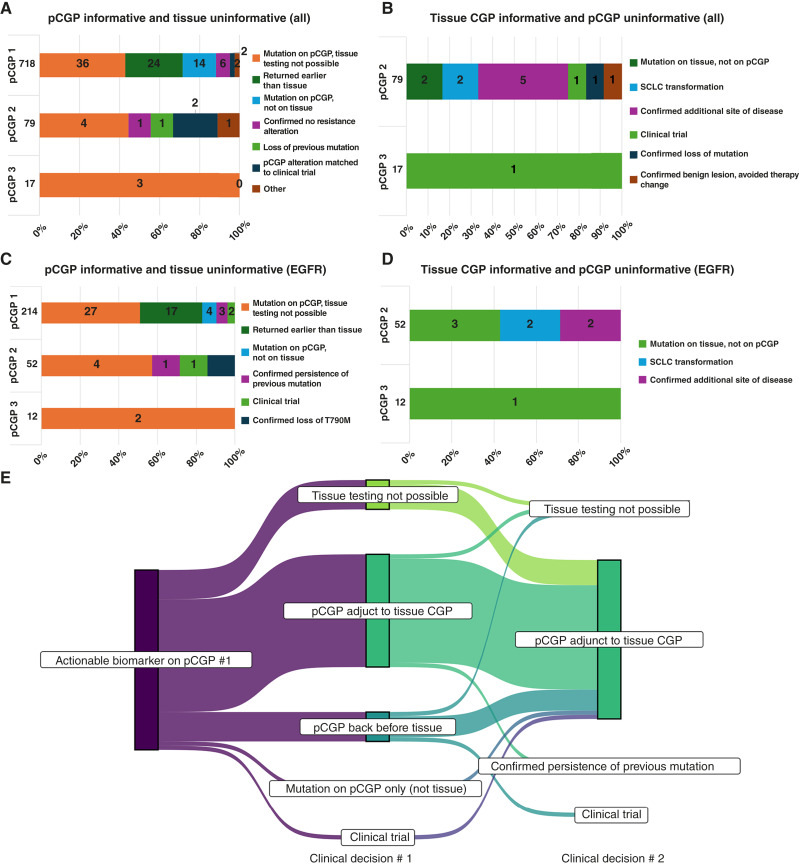
Impact of plasma genotyping vs. tissue on patient management. **A–D,** We considered circumstances in which pCGP was informative and concurrent tissue biopsy uninformative and then the converse, situations in which tissue biopsy was informative and concurrent pCGP uninformative. This latter analysis was limited to second and third assays of genotyping. The total number of patients with tissue biopsy was 31, of whom 22 (71%) had tissue NGS performed as well. Tumor fraction was not calculated as part of the pCGP assays. **A,** Instances when pCGP was informative and tissue uninformative, clustered according to the iteration of pCGP. For two patients marked as “other,” one had imaging changes that could represent NSCLC recurrence or *Mycobacterium avium* infection. The patient was hesitant to proceed with tissue biopsy, and the treating physician ordered pCGP, which identified a *KRAS* G12R mutation. This was considered to increase the likelihood of NSCLC recurrence and to justify the risks of tissue biopsy to obtain confirmation. In the second case, a frail patient had a *BRAF* V600E alteration on tissue NGS at first diagnosis and progressed through several lines of chemo- and immunotherapy. The treating physician performed repeat pCGP at progression and found no *BRAF* V600E. Given the toxicities of therapy and concern that the BRAF alteration was no longer present and circulating, the physician was guided not to pursue combined BRAF/MEK inhibition. **B,** Instances when tissue was informative and contemporaneous pCGP uninformative. **C,** In the *EGFR*m population subset, instances when pCGP was informative, whereas tissue NGS was uninformative. **D,** In the *EGFR*m population subset, instances when tissue was informative and contemporaneous pCGP uninformative. Although the number of cases was relatively small (*n* = 15), we found that concurrent tissue biopsy could guide management in more than half of the cases (57%) through information not available on pCGP, including histologic confirmation of radiographically equivocal sites of progression and the diagnosis of small cell histologic transformation. **E,** Sankey diagram illustrating the mechanisms through which pCGP influenced patient management at the first and second iterations of testing. pCGP was uniquely informative to patient management in the first and second iterations of testing, with benefits accruing to some patients more than once. At the first testing, it outperformed alternative methods for practical reasons, such as rapid turnover and permitting testing in patients who might not otherwise be fit, but it also identified some alterations that were not present on tissue NGS. It continued to be beneficial in the second iteration for patients in which tissue testing was not possible, which may reflect declining fitness for biopsy in this population.

We sought to assess the impact of pCGP in the management of patients with NSCLC (Fig. 1A–E; Supplementary Table S4). We considered a range of mechanisms by which pCGP could inform management beyond tissue findings, including earlier identification of mutations than with tissue genotyping, identification of mutations not detected by tissue genotyping, and enabling genotyping when a tissue biopsy was not feasible. Among those 718 patients on the first pCGP assay, in 84 cases (12%), the management decision was uniquely guided by pCGP results (Fig. 1A). Results of pCGP also uniquely guided benefit to patients on their second (10%) and third (18%) assays of genotyping. Overall, management was uniquely informed by any pCGP in 92 of 718 patients (13%). For three of 17 patients who had three serial pCGP assays (18%), their management changed on two separate occasions based on pCGP findings. Among 11 patients whose management changed on subsequent genotyping, 9 (82%) had *EGFR*m NSCLC. As an example, for one patient, the detection of an *EGFR* T790M mutation by pCGP allowed early matching to osimertinib and later detection of an *EGFR* C797S resistance mutation guided enrollment in a clinical trial. In another case, a patient who was too frail for tissue biopsy commenced gefitinib after pCGP identified an activating *EGFR* mutation; subsequent pCGP at progression informed a treatment change to osimertinib. In a third case, a patient was started on erlotinib early, based on pCGP findings, before tissue NGS, and was later matched to osimertinib based on pCGP identification of an *EGFR* T790M mutation.

In assessing cases where tissue testing was informative while pCGP was not, we performed a small subset analysis of cases with tissue testing (*n* = 31) or tissue NGS (*n* = 22) concurrent with pCGP (Supplementary Table S5). Turnaround time (TAT) for tissue versus plasma NGS in this subset was significantly shorter for plasma than for tissue NGS (7 vs. 15 days, *P* < 0.001). In these cases, concurrent tissue biopsy guided management in 42% of cases in which pCGP did not capture small cell transformation or small volume/radiographically equivocal sites of progression (Fig. 1B). Next, we identified patients within this group who had variants identified on pCGP but not on simultaneous tissue NGS (*n* = 11 patients, Supplementary Fig. S6). In total, 61 variants were uniquely detected in pCGP in these cases, of which 16 (26%) were characterized as oncogenic and 14 (23%) were characterized as likely oncogenic. The oncogenicity of the remainder was unknown (*n* = 29, 47%) or inconclusive (*n* = 2, 3%). Among genes canonically mutated in clonal hematopoiesis, eight alterations (13%) fell within this group: *CHEK2* R137Q (unknown oncogenicity), *ATM* R337C (likely oncogenic), and six alterations in *TP53*, of which five were likely oncogenic [Q192* (detected twice), E224D, G245D, and P152T] and a splice-site variant of unknown significance. In 2 of 14 (14%) patients, actionable mutations conferring TKI resistance were only detected in pCGP (*EGFR* C797S, *MET* D1228N). These findings suggest that variants uniquely detected in pCGP extend beyond clonal hematopoiesis and include actionable drivers of targeted therapy resistance.

### Liquid biopsy–informed management of oncogene-driven NSCLC

We then examined the impact of pCGP on clinical management in the subset of patients with *EGFR*m NSCLC (Fig. 1C and D; Supplementary Tables S6 and S7). In 28% of patients, pCGP uniquely guided patient management, benefiting both newly diagnosed patients and those undergoing second- or third-plasma genotyping. This benefit arose primarily from enabling the identification of actionable alterations without requiring tissue biopsy. Most commonly, pCGP was informative by identifying on-target resistance alterations in patients with *EGFR*m disease, whether T790M guiding a switch from first/second generation (1G/2G) TKI to third generation (3G), or C797S, prompting a switch from osimertinib to chemotherapy (Fig. 1C). pCGP also guided therapy by detecting off-target alterations in *BRAF* and *MET*. Overall, pCGP informed serial clinical decisions in the context of assay rapid TAT, feasibility of testing in cases where a tumor biopsy was not possible, and more optimally capturing tumor heterogeneity and evolution (Fig. 1E).

**Figure 2.**
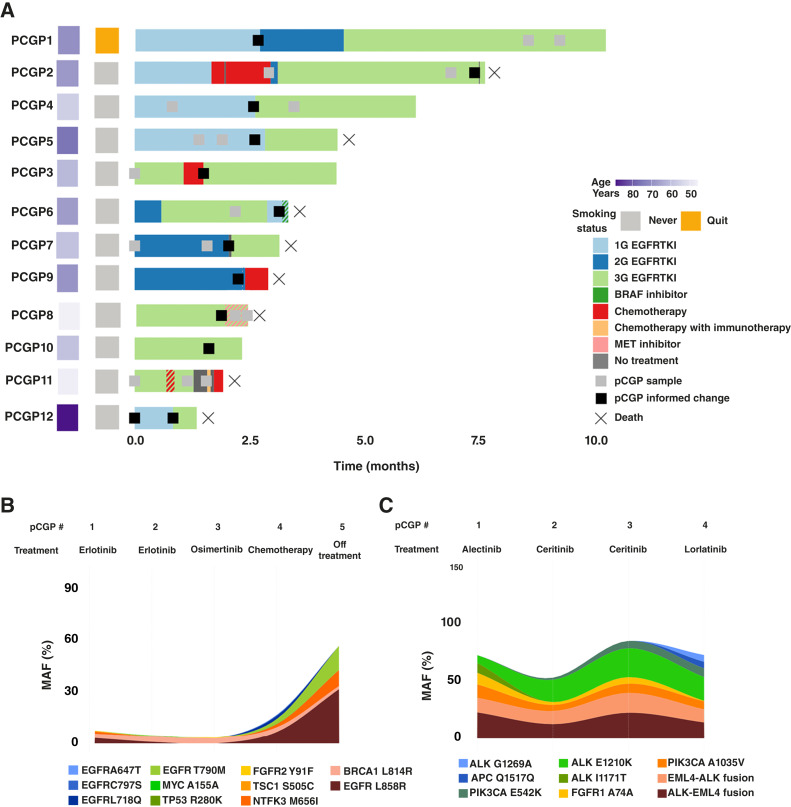
Impact of pCGP and ctDNA dynamics on patient management. **A,** Swimmer’s plot illustrating the survival of 12 patients with *EGFR*m NSCLC; these patients were chosen to provide a representative example of situations in which pCGP beyond the first diagnosis was informative for patient management. Examples of how pCGP informed management include the identification of T790M alterations (patients PCGP1, PCGP4, PCGP5, PCGP7, PCGP12); identification of a *BRAF* alteration (PCGP6); identification of *MET* amplification (PCGP8 and PCGP9); and identification of a C797S resistance alteration (PCGP2). **B** and **C,** The emergence of resistance on pCGP; fish plots illustrate a dynamic, longitudinal representation of molecular alterations identified on pCGP in patients with serial sampling. The *Y* axis represents the aggregate VAF of all alterations identified at each time point. Each vertical dashed line represents the time point of a pCGP assay, with the number beneath representing the VAF of the most prevalent alteration at that time. **B,** pCGP serial profiling for a patient with *EGFR* L858R–driven NSCLC who had five iterations of pCGP. They were on erlotinib up to the second pCGP, which was obtained at the time of radiographic progression. This identified *EGFR* T790M, and the patient was switched to osimertinib with subsequent suppression of this clone. The third pCGP was at the time of further radiographic progression, no new driver of resistance was identified but *EGFR* C797S was identified on liver biopsy. They were transitioned from osimertinib to a clinical trial at this point, with corresponding re-expansion of T790M and L858R variants after. Fourth pCGP, after carboplatin/pemetrexed/bevacizumab therapy, identified both C797S and L718Q, an uncommon mutation in *EGFR* and an additional resistance mechanism. Their final pCGP demonstrated a significant increase in the aggregate VAF of all alterations, and the patient died 2 months later. Separate regimens are separated by semicolons. **C,** pCGP serial profiling for a patient with *ALK*-fusion NSCLC that had four iterations of pCGP. Their disease had become resistant to alectinib at the time of the first pCGP, likely attributable to *ALK* E1210K and I1171T alterations. I1171T is associated with sensitivity to ceritinib, and the patient was switched to ceritinib at this time. After some clinical response with a corresponding contraction of the I1171T variant, expansion of the E1210K variant was seen in addition to an acquired oncogenic *PIK3CA* resistance variant. The third pCGP is at the time of progression on ceritinib. The patient transitioned to brigatinib initially but progressed after 6 months and transitioned to lorlatinib. Fourth pCGP after 22 months on lorlatinib identified an additional acquired on-target mutation in *ALK* G1269A. Compound mutations of this kind are common in patients on lorlatinib. Separate regimens are separated by semicolons.

Focusing on a subset of 12 patients with *EGFR*m NSCLC with serial pCGP assessments, pCGP past first diagnosis guided clinical management through the detection of *EGFR* T790M and C797S on-target resistance mutations, as well as off-target genomic alterations in *MET* and *BRAF* (Fig. 2A). As a representative example, for a patient with *EGFR*m NSCLC, five serial pCGPs identified three distinct mechanisms of resistance and a guided change in treatment, together with providing a real-time measurement of the circulating tumor burden (Fig. 2B). Similarly, a patient with *ALK* fusion–driven NSCLC had four pCGP assays while on targeted therapy, which led to the identification of four separate resistance variants and guided treatment changes twice (Fig. 2C). Some of these variants, such as *ALK* E1210K, were identifiable at a low variant allele fraction (VAF) in advance of a later increase in VAF and clinical progression. Taken together, in our study cohort, pCGP guided management decisions at both initial diagnosis and progression. These findings highlight the value of serial pCGP to identify actionable drivers of therapy resistance.

### Serial pCGP can identify actionable drivers of TKI resistance in *EGFR*m NSCLC

**Figure 3.**
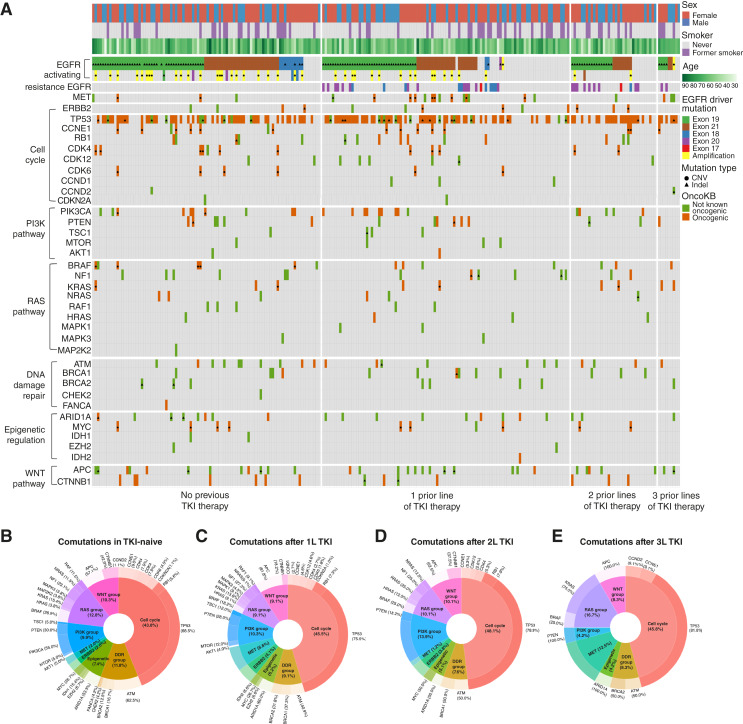
Comutations on pCGP in *EGFR*m NSCLC. **A,** Mutational landscape of *EGFR*m NSCLC in pCGP. Landscape figures represent the distribution of mutations among patients with *EGFR*m NSCLC, with findings clustered according to the number of previous lines of therapy. Each column represents an iteration of pCGP, and genes are clustered in rows according to the cellular pathway affected. Clinical metadata are represented in rows at the top of the diagram. The feature “EGFR activating” reflects *EGFR* mutations detected on pCGP assays. Several patients did not have *EGFR* mutations identified on pCGP but were included in this set as they were known to harbor *EGFR* activating mutations based on prior tissue NGS. We noted an early emergence of pathogenic alterations in genes in the PI3K pathway, late emergence of genomic alterations in the RAS pathway, and frequent cell-cycle gene alterations across settings. **B–E,** Key comutations in patients with *EGFR*m NSCLC by line of therapy. Pie–donut charts reflect the prevalence of *EGFR* comutations by the number of previous lines of TKI therapy. The inner circle in each chart reflects the cancer hallmark and gene families affected. The outer circle describes the individual genes altered. *MET* alterations are seen with increased frequency with later lines of therapy, whereas PI3K group alterations—predominantly *PTEN* mutations—become less frequent. DDR, DNA damage response.

We further focused on the subset of patients with *EGFR* alterations and studied patients with an actionable *EGFR* alteration identified on pCGP or who had such an alteration previously identified on tissue NGS (Fig. 3). We performed longitudinal tracking of mutant allele fractions (MAF) to understand tumor evolution under the selective pressure of EGFR-targeted therapies in the *EGFR* mutant subset. We used the maximal MAF (maxMAF) per pCGP to estimate the circulating tumor burden at each time point. Canonical clonal hematopoiesis mutations and germline polymorphisms were filtered out before calculating maxMAF. We assessed maxMAF across all variants, then the MAF of driver *EGFR* alterations. We did not detect any differences in maxMAF between pCGP1 and pCGP2, pCGP1 and pCGP3, or pCGP2 and pCGP3 (Wilcoxon rank-sum test *P* = 0.76, *P* = 0.33, and *P* = 0.25, respectively). Subset analyses of paired samples revealed similar findings. Next, we focused on MAF differences of *EGFR* driver mutations across pCGP time points. Paired analyses showed a lower driver *EGFR* mutation MAF in the second instance of pCGP (paired *t* test *P* = 0.048), consistent with the clearance of cancer clones under the selective pressure of EGFR TKI therapy.

In addition to patients with *EGFR* canonical alterations in exons 19 (55%) and 21 (34%), a small number of patients had mutations in exon 18 (9%) and *de novo* exon 20 T790M mutations (1%; Supplementary Fig. S4). Of note, we excluded patients with oncogenic *EGFR* exon 20 insertions due to their unique natural history, resistance to EGFR TKIs and lack of actionable guideline-directed therapy during the study period. Among patients with otherwise sensitizing *EGFR* alterations (*n* = 214), there were 280 instances of pCGP across 0 to 3 previous treatment lines. This subset of patients were notable for having a greater proportion of patients who were female, never smokers, and of Asian ethnicity and were composed almost entirely of adenocarcinomas (Table 1). Of those whose treatment histories were recorded, nearly equal numbers received upfront treatment with a 1G or 2G EGFR TKI (*n* = 73, 52%) or 3G TKI (*n* = 67, 48%).

We first evaluated the emergence of on-target resistance mutations (Supplementary Table S8). In the subset of patients receiving a 3G EGFR TKI, we found emerging C797S mutations after first-line treatment (1LT; 4 patients of 67, 6%), with 3 of 4 (75%) patients with C797S harboring compound mutations with L718Q (*n* = 2) and G724S (*n* = 1), respectively (Fig. 3A). In addition, 10 patients acquired mutations in the *EGFR* kinase domain after TKI therapy, which were not previously recognized as resistance alterations but had computational characteristics suggestive of oncogenicity (Supplementary Table S9).

We then sought to characterize the landscape of EGFR comutations in the overall population and in subsets by line of therapy (Fig. 3A). Cell-cycle alterations were the most common comutation with *EGFR* and were identified frequently at treatment-naive baseline (54/98 patients, 55%) but also after subsequent therapy (57/109 patients after 1LT, 52%; Fig. 3B). We identified co-occurrence of *EGFR* amplifications with *EGFR* sensitizing alterations in 23 of 98 treatment-naïve patients (23%) and 11 of 109 (10%) patients after 1LT TKI. Eight patients had oncogenic *RB1* alterations across time points, of whom 3 (37.5%) had tissue-confirmed small cell transformation. In two of these patients, the *RB1* alteration was not present on earlier pCGP but was detectable on pCGP at the time of tissue biopsy. We further explored off-target resistance according to the first TKI received (1G or 2G vs. 3G; Fig. 3B–E; Supplementary Fig. S5; Supplementary Table S10). After 1LT, the most frequent resistance mechanism was via alterations in *MET* for patients in the 1G or 2G group (*n* = 3, 8% of all patients) and *PIK3CA* for patients in the 3G group (*n* = 7, 11%; Fig. 3C). However, after 3LT, resistance in the 1G or 2G group was more common via alterations in *KRAS* (*n* = 2, 22%), with select patients having mutations in *BRAF* (*n* = 1, 11%) and *MET* (*n* = 1, 11%; Supplementary Table S10; Fig. 3E).

The gene most frequently involved in off-target resistance was *PIK3CA*. When examining all patients with *PIK3CA* alterations across all lines, these were found predominantly in the helical domain in both the 3G (5/7, 71%) and 1G or 2G groups (4/6, 67%). Oncogenic mutations in *PTEN* were also noted in four (6%) patients in the 3G group and two (3%) in the 1G or 2G group. These alterations were only in the dual specificity protein phosphatase domain in the 3G group (4/4, 80%) but only in the C2 domain in the 1G or 2G group (2/2, 100%).

Focusing on MAPK pathway alterations, *KRAS* alterations in codons 12 or 61 were identified in 1G/2G patients (4%) in the second- or third-line setting (Fig. 3D and E). Two of sixty-seven patients who had received 3G TKI (3%) acquired an oncogenic *BRAF* V600E mutation. Notably, both of these patients subsequently developed small cell transformation in the absence of *RB1* comutation. *MET* amplifications were identified at similar frequencies in the 1G or 2G group (*n* = 4, 5%) and 3G group (*n* = 4, 6%). Of 26 patients with paired pCGP at baseline and after TKI, 3 (12%) had new *MET* amplifications. However, exon 14 skipping mutations were identified only in the 3G group (2 out of 67, 3%).

Of note, of the 30 *EGFRm* patients who had concomitant tissue biopsy with pCGP after 1L TKI, 5 (20%) were found to have *MET* amplifications in tissue. *MET* amplification was not detected in any of these cases on concurrent pCGP. This discordance may reflect technical challenges of tumor-naïve hybrid capture NGS for structural alteration detection in the context of low circulating tumor burden. In line with this notion, two patients with discrepant tissue and plasma CGP had low-burden/thoracic disease only. Notably, among 79 patients with *EGFR*m NSCLC who had pCGP at progression on 1L TKI but did not have concomitant tissue biopsy, 7 (9%) were found to have a *MET* amplification, supporting the clinical utility of pCGP in capturing *MET*-driven TKI resistance in cases where tissue biopsies are not feasible.

Overall, 24 (18%) patients with pCGP during treatment had results revealing acquired resistance mutations with an FDA-approved therapy in NSCLC. When amplifications in *ERBB2* and *MET* were included, 31 (22%) patients had actionable pCGP findings on progression; 18 of these 31 patients (58%) were matched to therapy based on pCGP. No oncogenic fusions were identified on pCGP as acquired drivers of resistance to EGFR TKI, likely due to the limited sensitivity of liquid biopsies for fusion detection. Our findings highlight how resistance pathways in *EGFR*m NSCLC differ according to the sequence of therapies and demonstrate how pCGP enables the initiation of new therapies tailored to acquired resistance alterations.

### Association between pCGP variants and survival

**Figure 4.**
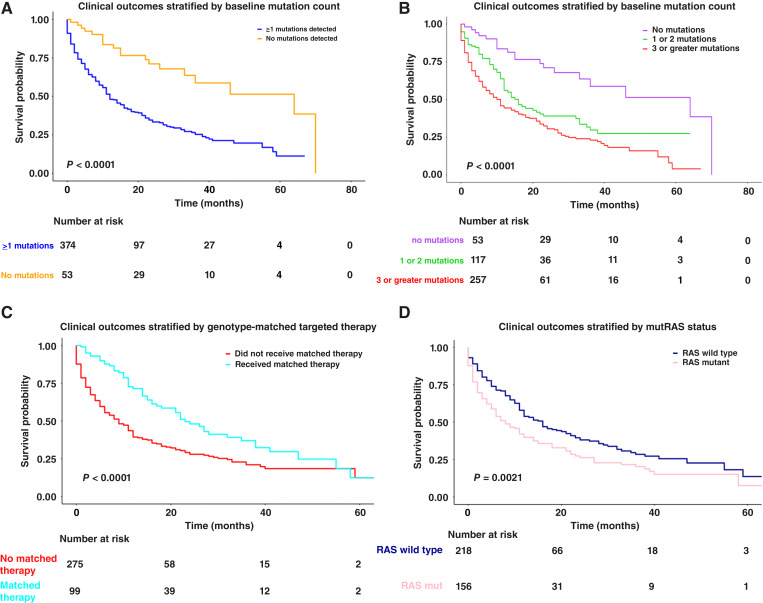
Association between pCGP parameters and OS. **A,** Kaplan–Meier survival curves for patients with no alterations identified on pCGP vs. any alteration identified on pCGP. **B,** Kaplan–Meier survival curves for patients with three or more mutations on pCGP or fewer than three mutations. **C,** Kaplan–Meier survival curves for patients who received targeted therapy matched to an alteration on pCGP and those who did not receive a matched therapy. **D,** Kaplan–Meier survival curves for patients who had alterations in the RAS pathway or those who had no RAS pathway alteration. *P* values are provided using the log-rank test; of note, comparisons in **C** and **D** did not include patients with zero alterations detected by pCGP.

We then explored whether any findings on baseline pCGP were associated with OS, with analysis limited to the first instance of pCGP (treatment-naïve setting; *n* = 427). Patients with no alterations identified on baseline pCGP had significantly prolonged OS compared with those with ≥1 mutations detected (log-rank *P* < 0.0001; Fig. 4A), with similar results when patients were categorized as ctDNA undetectable, 1 to 2 detected mutations, and ≥3 detected mutations (log-rank *P* < 0.0001; Fig. 4B).

We next focused on the subset of patients who received targeted therapy with an FDA-approved agent for an alteration detected on the first pCGP and compared their outcomes with patients who had detectable alterations on the first pCGP but did not receive genotype-matched therapy. Receipt of genotype-matched therapy was associated with a significant improvement in survival (log-rank *P* < 0.0001; Fig. 4C). We then examined the association between alterations in specific gene pathways on baseline pCGP and outcomes, limiting the analyses to pCGP at first diagnosis. We categorized genes according to their role in cancer hallmarks and pathways: cell cycle, DNA damage repair, PI3K/AKT/mTOR, RAS/RAF/MAPK, epigenetic, WNT signaling, and tumor suppression. Notably, patients with any alteration in the RAS pathway had inferior survival compared with those without RAS alterations (log-rank *P* = 0.002; Fig. 4D). Lastly, given the frequency of *TP53* mutations in *EGFR*-mutant NSCLC, we assessed the association between *EGFR* and *TP53* comutations in this patient subset. Although *TP53* comutations were not associated with altered survival among patients with *EGFR* mutations (log-rank *P* = 0.35), disruptive *TP53* mutations were associated with inferior survival in patients with *EGFR* wild-type NSCLC (log-rank *P* = 0.043). Taken together, our findings point toward the prognostic value of ctDNA levels and genotypes for patients with metastatic NSCLC.

## Discussion

We conducted this study to assess the prevalence of actionable alterations detected with pCGP in NSCLC and their impact on management, with a focus on *EGFR*m disease. We found that pCGP can capture actionable driver alterations and putative drivers of resistance, and this enables clinical utility for management across diagnosis and longitudinal care. In addition, certain parameters on baseline pCGP, such as the presence of RAS alterations or undetectable ctDNA, were associated with differential survival.

Although the prevalence of actionable alterations is dependent on ancestry and environmental exposures (32), the detection of alterations associated with an FDA-approved targeted therapy in 36% of patients at first diagnosis in our cohort is comparable with previous studies (15, 17). Among those with sensitizing alterations in *EGFR* and *ALK* on pCGP at first diagnosis, representing the most common alterations for which there is an indication for FDA-approved therapy in the first-line setting, 93% of patients commenced a matched therapy within 30 days of pCGP results. Emerging alterations that may be actionable but do not yet have FDA approval for targeted therapy, such as copy-number variations in *MET* and *ERBB2*, were also evident, reinforcing the role of genomic profiling through liquid biopsies.

With respect to treatment resistance in *EGFR*m disease, we identified on-target alterations occurring both in variants previously recognized (such as *EGFR* C797S) and in novel sites with computational characteristics suggestive of pathogenicity. These included variants in *EGFR* exon 18 (K714E), exon 19 (K754), and exon 21 (V834L), for which bioinformatic assessment of functional consequence generated a high probability of oncogenicity. Notably, more than 50% of *EGFR* C797S mutations arose as compound rather than single mutations. These findings are in line with previous literature suggesting more heterogeneous mechanisms of on-target resistance to 3G EGFR TKIs compared with 1G or 2G agents (33). These disparate mechanisms pose a clinical challenge because they are not amenable to a uniform approach to clinical management. However, the rapid identification and structure-based classification of variants to predict drug resistance and response represent a promising avenue for addressing TKI resistance in *EGFR*m disease (34).

More than half of the patients in our study with actionable findings on disease progression were subsequently matched to targeted therapy, supporting the clinical utility of liquid biopsies to guide therapy selection when resistance develops. The presence of actionable alterations at progression in 22% of patients with *EGFR*m tumors is noteworthy, as it may open options beyond cytotoxic chemotherapy. Although several drivers of off-target resistance were shared among the 1G or 2G and 3G patient groups, the 3G group had unique alterations associated with an FDA-approved therapy, including *MET* exon 14 skipping mutations. Oncogenic *BRAF* V600E alterations were found more commonly in the 3G group and only after later-line therapy in the 1G/2G group. It also seemed that some genes were more commonly altered after 1LT, such as in the PI3K pathway, whereas others were more commonly altered in the later-line setting, such as the RAS group. The identification of *RB1* mutations in patients with small cell transformation demonstrates the ability of pCGP to detect molecular markers of evolving tumor biology. These findings may allow earlier tissue biopsy to confirm diagnosis and guide a change in treatment. Overall, the most notable finding about resistance alterations seems to be the differential activation of pathways according to treatment context: the first TKI received and the line of therapy. As the range of treatment options for oncogene-addicted tumors expands, our findings support a role for serial pCGP to guide management upon the emergence of therapy resistance.

Previous evidence suggests that early identification of resistance alterations on pCGP can improve clinical outcomes. In the APPLE trial, switching from a 1G to a 3G EGFR TKI—prompted by the detection of the *EGFR* T790M mutation in ctDNA before radiographic disease progression—proved feasible and resulted in a progression-free survival benefit (13, 35). Similar findings have been observed in patients with breast cancer who switched from an aromatase inhibitor and palbociclib to fulvestrant and palbociclib after detecting *ESR1* mutations in ctDNA. The ctDNA-guided therapy switch has demonstrated significant clinical benefits in the PADA-1 clinical trial (36). Likewise, the SERENA-6 study found that regenotyping ctDNA to identify emerging *ESR1* mutations and then randomizing patients to camizestrant with ongoing CDK4/6 inhibitor treatment improved progression-free survival in the ctDNA-informed group (37).

Data remain limited about the clinical impact of serial pCGP in the management of NSCLC. We found that pCGP can uniquely inform management beyond tissue-based testing for 13% of patients, and this benefit accrues across the first, second, and third pCGP iterations. This demonstrates the potential utility of pCGP in routine practice at both initial diagnosis and progression. Nevertheless, tissue biopsy will remain a necessity for specific patients with concerns about small cell transformation, low-shedding tumors, or uncertainty about disease progression. Importantly, discordance between tissue and plasma CGP may reflect technical challenges of tumor-naïve hybrid capture NGS in the context of low circulating tumor burden close to or below the limit of detection of a given liquid biopsy assay, especially when structural genomic alterations are considered (6, 11, 38).

Consistent with previous studies, we found a significant association between undetectable ctDNA and improved OS (20). This phenomenon is likely due to a lower burden of disease and, correspondingly, better outcomes in patients with undetectable ctDNA. It is, however, not possible to distinguish between a true absence of circulating variants and the presence of variants below the assay’s limit of detection. Although a significant association between extrapulmonary disease and detectable ctDNA has been previously reported (20), in our study cohort, we lacked sufficient detail about the extent of intrathoracic disease and sites of metastasis to investigate this further. Receipt of FDA-approved therapy matched to a pCGP alteration was associated with improved survival. This is consistent with the high response rates and survival advantage associated with targeted therapies and reinforces similar findings from previously published data (20).

Disruptive alterations in *TP53*, which affect its DNA-binding domain, are biochemically predicted to have a more deleterious effect (39). There have been conflicting findings on their clinical impact in cancer, and even within lung cancer, both disruptive (40) and nondisruptive (41) alterations have been associated with inferior prognosis. We did not identify significant differences in survival between those with disruptive and nondisruptive mutations in the overall population or in the *EGFR*m subset; however, those with *EGFR* wild-type disease harboring a disruptive mutation had poorer survival than those with nondisruptive mutations.

Our work has several limitations. This study was retrospective, which limits the strength of its conclusions. Furthermore, only 11% of patients had two or more pCGP samples, which limits the population included in longitudinal analyses. In addition, there was heterogeneity in the context of pCGP (first diagnosis vs. metastatic progression), NGS assays used, and TKI therapies. A small number of our population had earlier-stage cancers, which represent a different clinical context than advanced NSCLC, although increasing evidence supports a role for pCGP in this setting, especially in *EGFR*- and *ALK*-driven NSCLC (42, 43). Lastly, the comparison of concurrent tissue and plasma NGS was performed only in a smaller subset of patients, which precludes firm conclusions.

In conclusion, pCGP can identify actionable driver alterations and drivers of therapy resistance in patients with NSCLC, particularly among those with *EGFR*m disease. The use of pCGP at first diagnosis and at the time of disease progression can inform patient management, reinforcing the critical role of plasma testing as a standard-of-care companion to tissue NGS. Rigorous characterization of variants emerging during therapy resistance is required to verify the role of an ever-growing list of alterations across the continuum of NSCLC and the clinical utility of liquid biopsies to guide therapy decision-making.

## Supplementary Material

Supplementary Figure S1 Figure S1. Landscape of all alterations identified by pCGP.

Supplementary Figure S2 Figure S2. Actionable alterations identified on all instances of first pCGP.

Supplementary Figure S3 Figure S3. Actionable alterations identified on first pCGP at first diagnosis (treatment-naive setting).

Supplementary Figure S4 Figure S4. Categorization of EGFR driver variants identified in the EGFRm NSCLC subset.

Supplementary Figure S5 Figure S5. Mechanisms of resistance to EGFR TKI according to first line of therapy received.

Supplementary Figure S6 Figure S6. Plasma-only variants among patients with simultaneous plasma and tissue NGS.

Supplementary Table S1 Supplementary Table S1. Context of first pCGP assays.

Supplementary Table S2 Supplementary Table S2. Cumulative mutation frequencies per gene and variant type detected on first pCGP.

Supplementary Table S3 Supplementary Table S3. Summary of genotype-matched therapies.

Supplementary Table S4 Supplementary Table S4. Description of pCGP-informed clinical management.

Supplementary Table S5 Supplementary Table S5. Summary of tissue informed clinical management in patients with concurrent pCGP and tissue biopsy.

Supplementary Table S6 Supplementary Table S6. pCGP-informed clinical management in EGFR-mutant NSCLC.

Supplementary Table S7 Supplementary Table S7. Tissue informed clinical management in EGFR-mutant NSCLC.

Supplementary Table S8 Supplementary Table S8. On-target acquired resistance in EGFR-mutant NSCLC.

Supplementary Table S9 Supplementary Table S9. Putative uncommon on-target drivers of EGFR TKI resistance.

Supplementary Table S10 Supplementary Table S10. Drivers of EGFR TKI resistance.

## Data Availability

The data generated in this study are not made publicly available to protect patient privacy but are available from the corresponding author upon reasonable request.
