# Supplementary figures and images for: Serial Plasma Comprehensive Genomic Profiling Captures Therapy Resistance and Guides Management of Non–Small Cell Lung Cancer
Source: Cancer Res Commun. 2026 May 21;6(5):1180–91. doi: 10.1158/2767-9764.CRC-25-0561 (PMC13192330; doi:10.1158/2767-9764.CRC-25-0561)

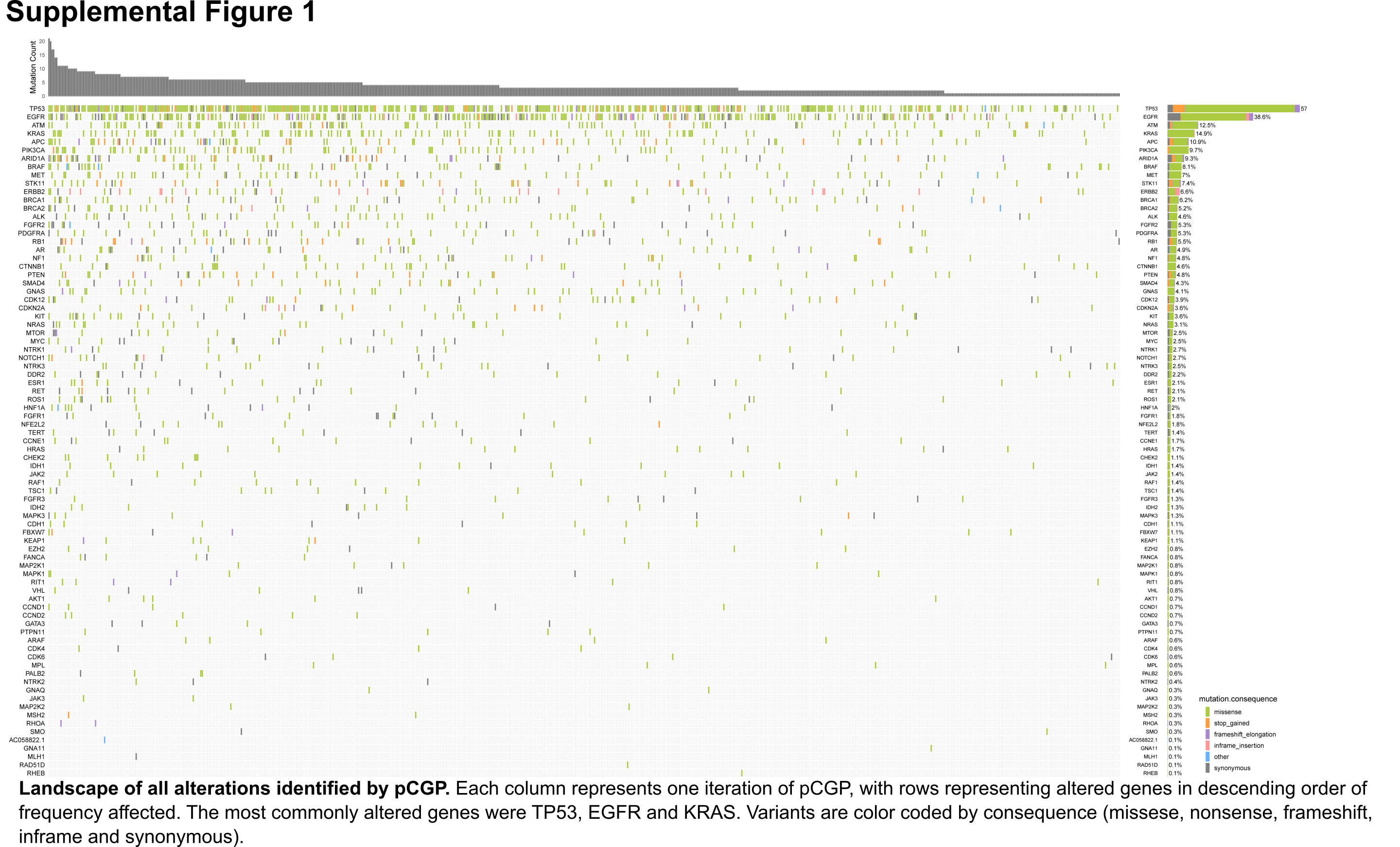

Supplement: Supplementary Figure S1 — Figure S1. Landscape of all alterations identified by pCGP. [file crc-25-0561_supplementary_figure_s1_suppsf1.png]

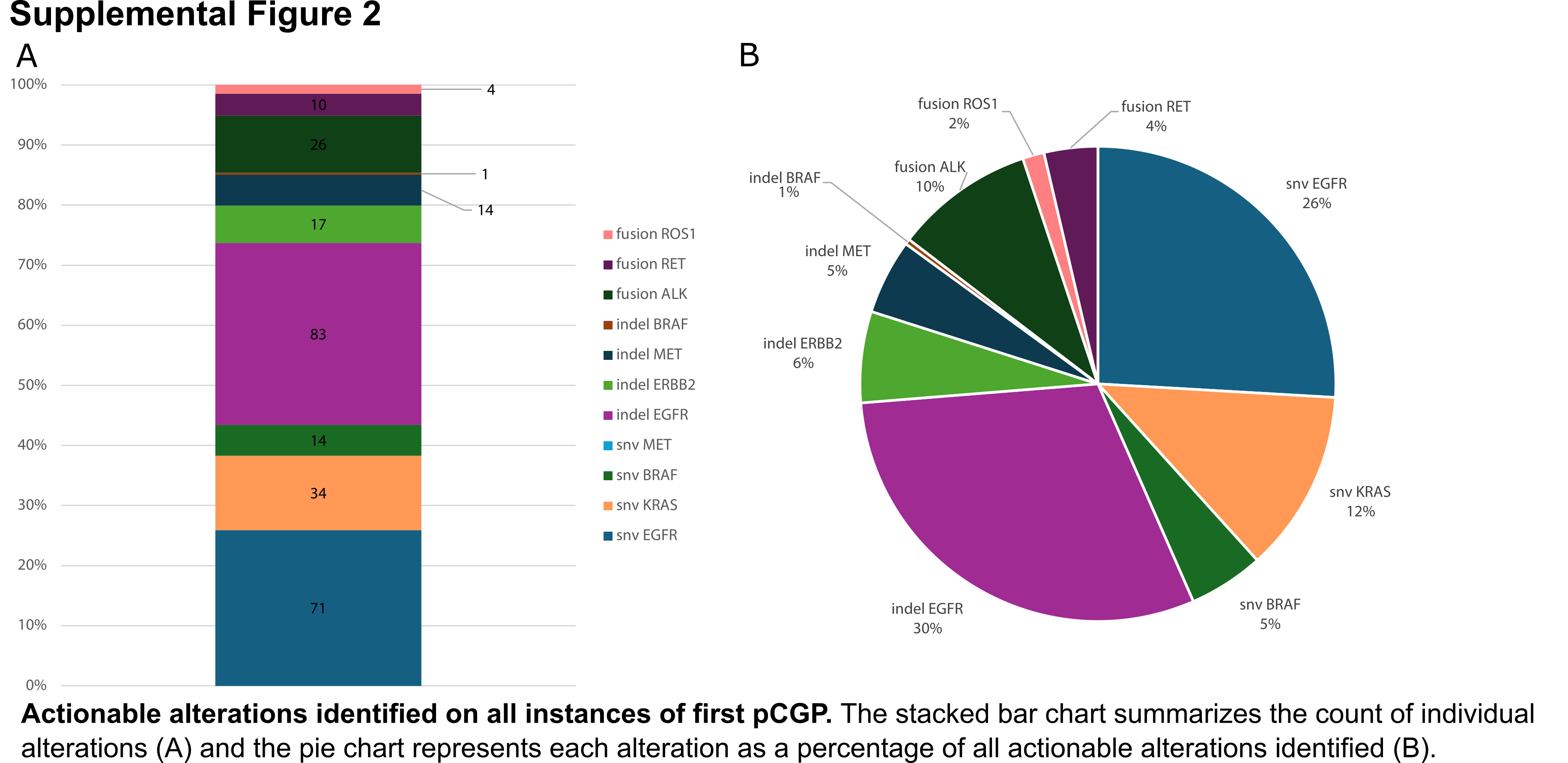

Supplement: Supplementary Figure S2 — Figure S2. Actionable alterations identified on all instances of first pCGP. [file crc-25-0561_supplementary_figure_s2_suppsf2.png]

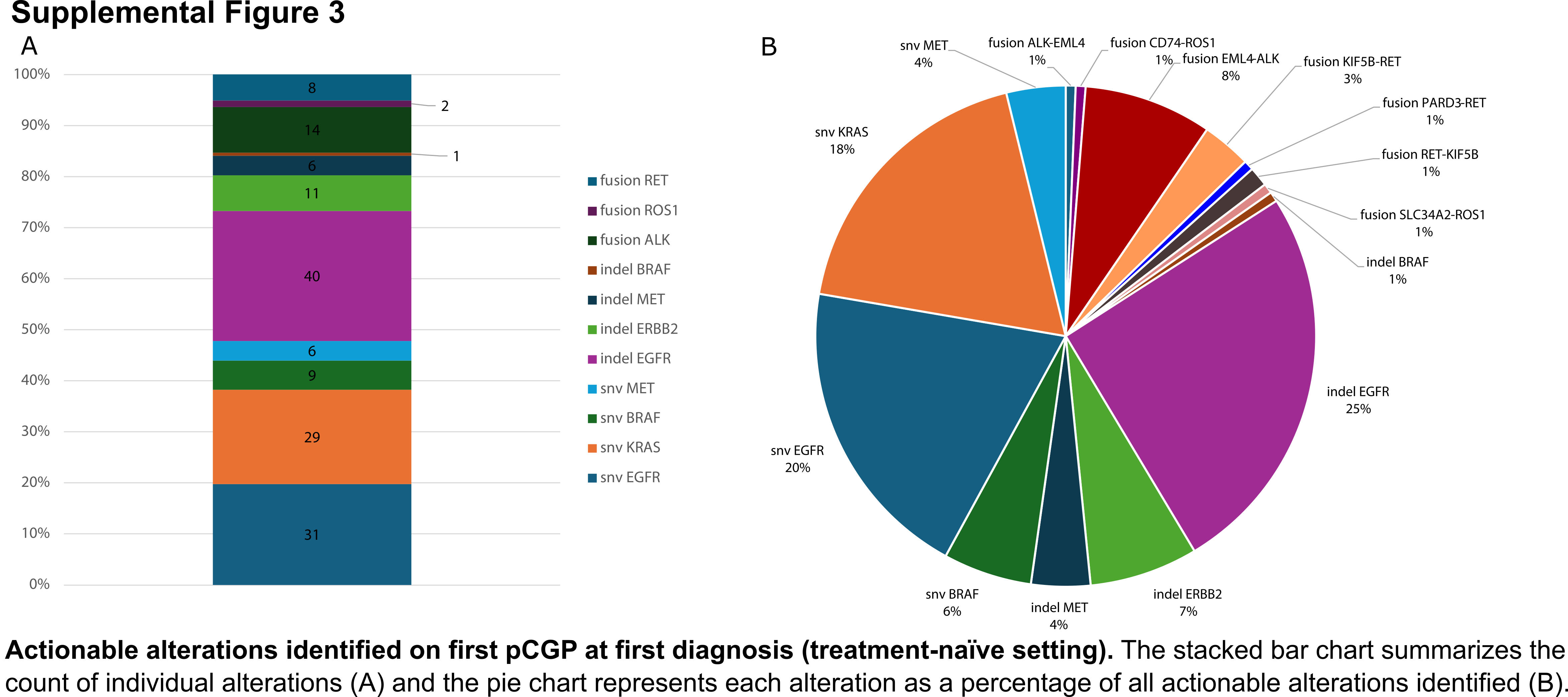

Supplement: Supplementary Figure S3 — Figure S3. Actionable alterations identified on first pCGP at first diagnosis (treatment-naive setting). [file crc-25-0561_supplementary_figure_s3_suppsf3.png]

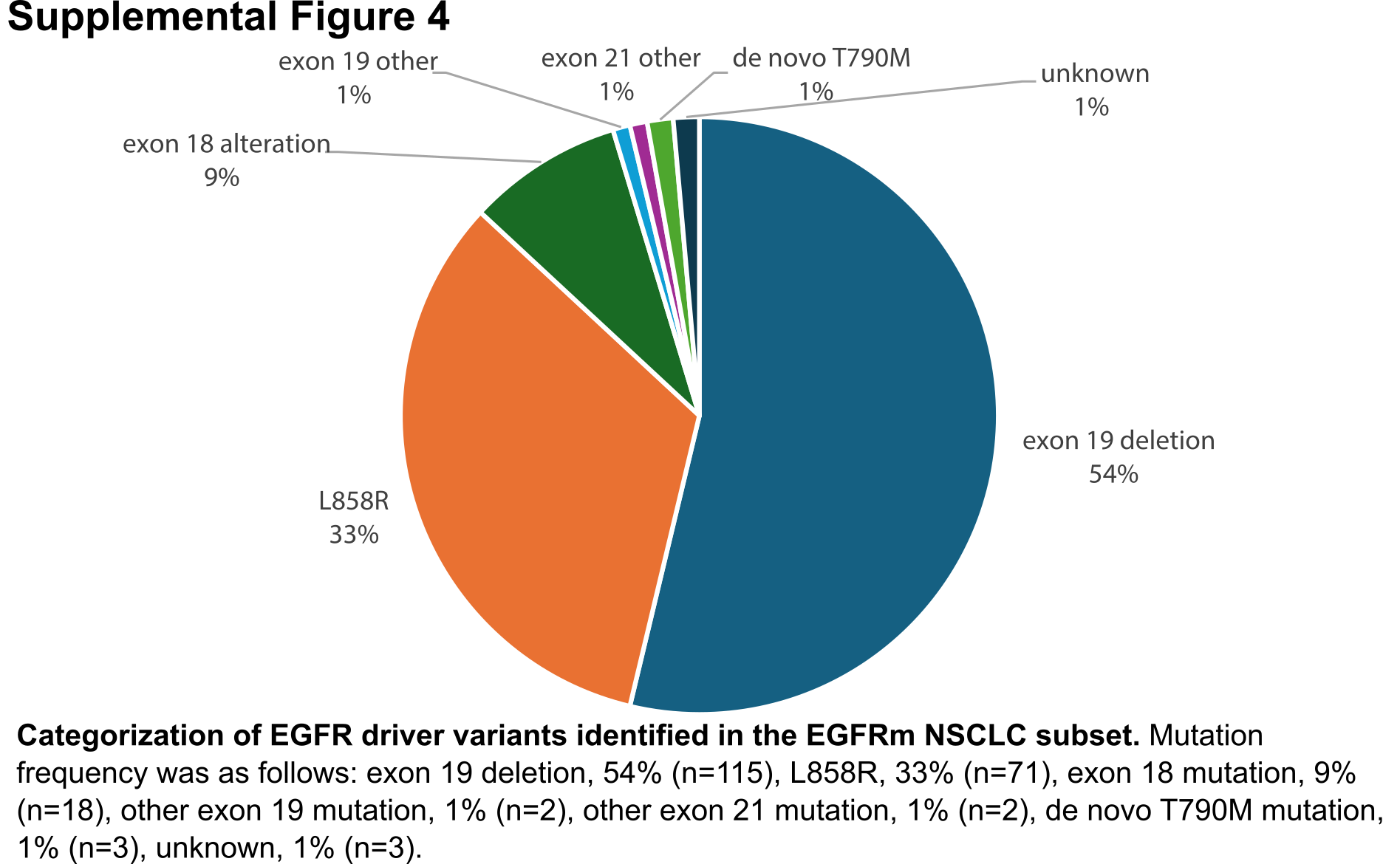

Supplement: Supplementary Figure S4 — Figure S4. Categorization of EGFR driver variants identified in the EGFRm NSCLC subset. [file crc-25-0561_supplementary_figure_s4_suppsf4.png]

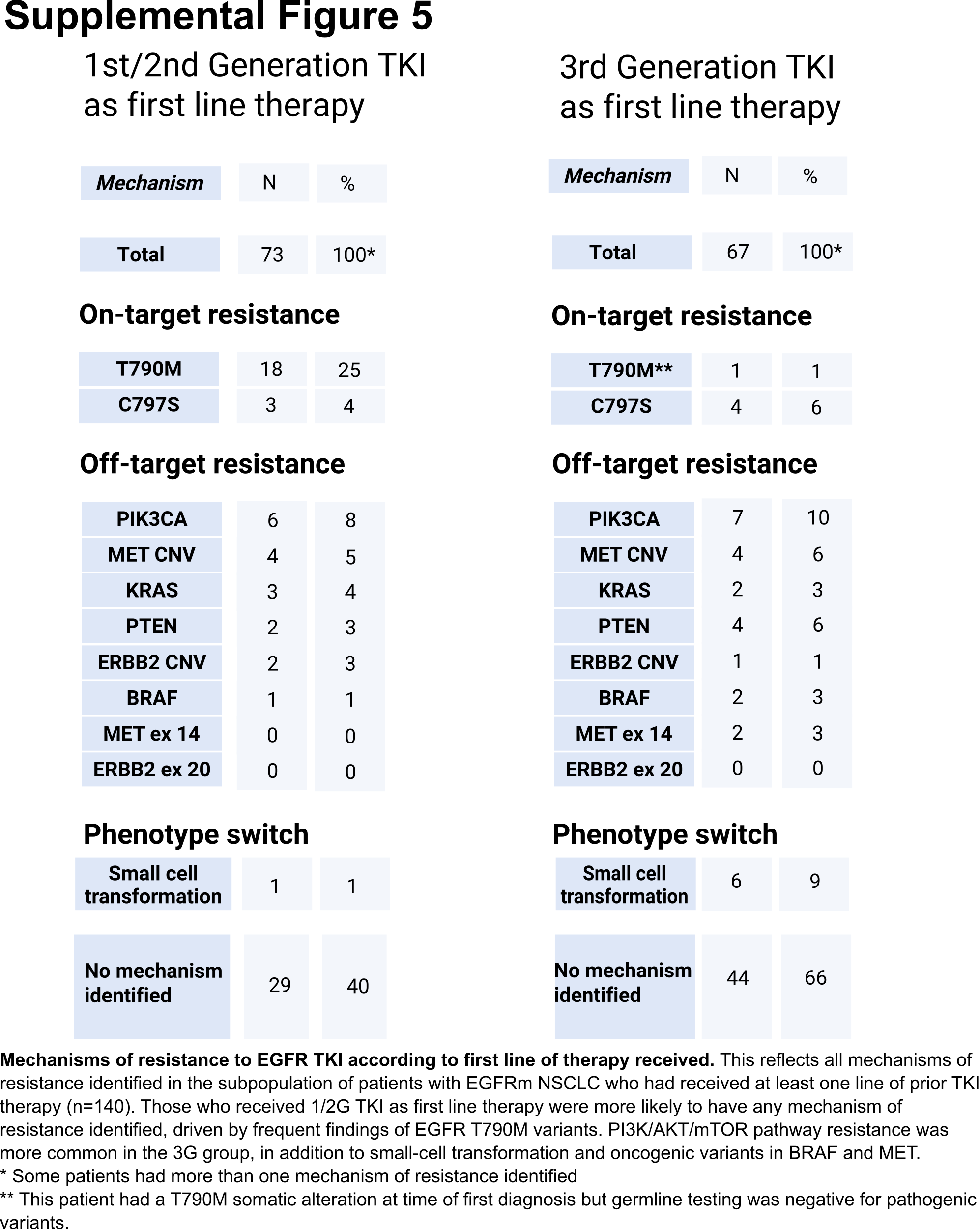

Supplement: Supplementary Figure S5 — Figure S5. Mechanisms of resistance to EGFR TKI according to first line of therapy received. [file crc-25-0561_supplementary_figure_s5_suppsf5.png]

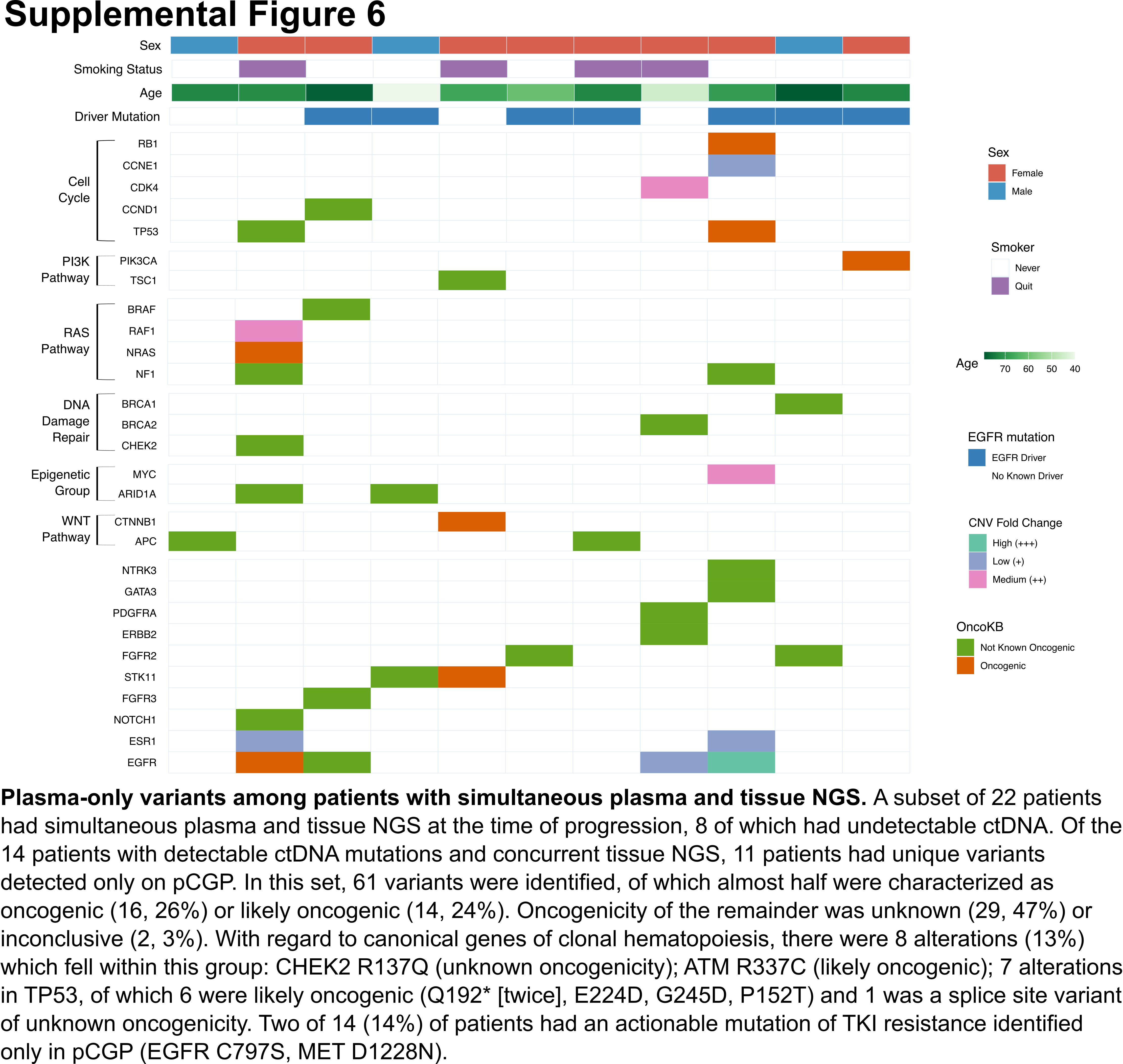

Supplement: Supplementary Figure S6 — Figure S6. Plasma-only variants among patients with simultaneous plasma and tissue NGS. [file crc-25-0561_supplementary_figure_s6_suppsf6.png]
